# The foot and ankle characteristics in children with idiopathic toe walking gait

**DOI:** 10.1186/1757-1146-6-S1-O39

**Published:** 2013-05-31

**Authors:** Cylie Williams, Paul Tinley, Michael Curtin, Sharon Nielsen

**Affiliations:** 1 Allied Health Research Unit, Southern Health, Cheltenham, VIC, 3192, Australia; 2 Department of Physiotherapy, Monash University, Frankston, VIC, 3199, Australia; 3 School of Podiatry, Charles Sturt University, Albury, NSW, 2460, Australia; 4 School of Occupational Therapy, Charles Sturt University, Albury, NSW, 2460, Australia; 5 Quantitative Consulting Unit , Charles Sturt University, Wagga Wagga, NSW, 2678, Australia

## Background

Idiopathic toe walking (ITW) has been associated with ankle equinus, and while equinus has been linked with foot deformity in adults, there has been limited investigation on its impact on structural foot change in children. This study used the weight bearing lunge (WBL) test and Foot Posture Index-6 (FPI-6) to evaluate the foot and ankle measures of children with an ITW gait.

## Methods

Sixty children between the ages of four and eight years were grouped into an ITW (N=30) and a non-toe walking (NTW) (N=30) cohort. The ankle range of movement and FPI-6 was calculated during appropriate weight–bearing test and stance.

## Results

There was a highly significant difference in the WBL test measures between the ITW cohort and the NTW cohort. The FPI-6 comparison was not significant. The WBL test was also not predictive of an abnormal FPI-6 in the ITW cohort.

## Conclusion

These results demonstrate that ITW gait style impacts only on the available dorsiflexion at the ankle. The WBL measure may be utilised within the clinical setting to guide and monitor treatment interventions.

